# Determinants of changes in women’s and men’s eating behavior across the transition to parenthood: a focus group study

**DOI:** 10.1186/s12966-021-01137-4

**Published:** 2021-07-12

**Authors:** Vickà Versele, F. Marijn Stok, Dirk Aerenhouts, Benedicte Deforche, Annick Bogaerts, Roland Devlieger, Peter Clarys, Tom Deliens

**Affiliations:** 1 Faculty of Physical Education and Physiotherapy, Department of Movement and Sport Sciences, Vrije Universiteit Brussel, Pleinlaan 2, 1050 Brussels, Belgium; 2 Faculty of Medicine, Department of Development and Regeneration, KU Leuven, Herestraat 49, 3000 Leuven, Belgium; 3 Department of Interdisciplinary Social Science, Utrecht University, Heidelberglaan 1, 3584 Utrecht, CS Netherlands; 4 Faculty of Medicine and Health Science, Department of Public Health and Primary Care, Ghent University, C. Heymanslaan 10, 9000 Ghent, Belgium; 5 Faculty of Medicine and Health Sciences, Centre for Research and Innovation in Care (CRIC), University of Antwerp, 2000 Antwerp, Belgium; 6 Faculty of Health, University of Plymouth, Plymouth, Devon PL4 8AA UK; 7 Obstetrics and Gynaecology, University Hospitals KU Leuven, Herestraat 49, 3000 Leuven, Belgium

**Keywords:** Pregnancy, Postpartum, Nutrition, Qualitative research

## Abstract

**Background:**

During the pregnancy and postpartum period, both women and men experience physiological and psychological changes, which may negatively impact their eating behavior. A clear understanding of determinants of changes in eating behavior during this period is needed to facilitate the development of targeted family-based interventions countering unfavorable dietary changes during this critical life period.

**Methods:**

Thirteen focus group discussions targeting determinants of changes in eating behavior during pregnancy and postpartum were conducted, involving a total of 74 expecting and first-time parents. A semi-structured question guide was used to facilitate the discussions. An inductive thematic approach was used to derive main and sub-categories of determinants from the data. The Determinants of Nutrition and Eating (DONE)-framework was employed to systematically organize and label the categories and determinants.

**Results:**

Two frameworks were developed; one for the pregnancy and one for the postpartum period, comprising determinants of changes in eating behavior in both women and men. Three main levels of determinants were identified: (1) the individual level, including psychological (e.g., ‘health consciousness’), situational (e.g., ‘effort and convenience’) and biological (e.g., ‘discomfort’); (2) the interpersonal level (e.g., ‘social influence’) and (3) the environmental level, including micro- and meso/macro (e.g., ‘home/environment food availability’). Determinants acting as barriers (e.g., ‘time constraints’) or facilitators (e.g., ‘being a role model’) were identified. Many determinants were mentioned during both (e.g., ‘food knowledge’) or just one investigated period (e.g., ‘physiological changes’ during pregnancy, ‘influence of the baby’ postpartum). Finally, some were described by both parents (e.g., ‘self-regulation’), whereas others were mentioned by women (e.g., ‘(perceived) food safety’) or men (e.g., ‘other priorities’) only.

**Conclusion:**

The developed frameworks set the foundation for the development of future family-based interventions and may be used already by healthcare providers to provide dietary guidance and support for women and men transitioning into parenthood. A focus on the interplay of individual factors at the biological and psychological level together with situational difficulties during pregnancy is recommended. Postpartum, focus should go to support first-time parents to obtain balance of both maintaining one’s own health and taking care of the baby, on improving self-regulation skills, and on coping with related situational constraints.

**Supplementary Information:**

The online version contains supplementary material available at 10.1186/s12966-021-01137-4.

## Background

Pregnancy and parenthood are lifechanging periods in many different aspects, for both mothers and fathers [1–4]. During pregnancy and postpartum, women experience physiological changes in terms of hormones, body weight and fat disposition and cardiovascular, respiratory and gastrointestinal functions [5–7]. This period is likewise challenging in several psychological (e.g., increased stress, emotional disturbance, worries and feelings of uncertainties) and behavioral ways, including changes in sleep, physical activity and eating behavior [1, 8–11]. Even though mothers are more vulnerable to experience these changes, for expecting and first-time fathers this too has been described as a stressful transition period in which they experience some levels of mental, physical and lifestyle changes [2, 5–12]. For women, these challenges may result in excessive gestational weight gain (GWG) during pregnancy and a postpartum body weight often not returning to pre-pregnancy levels [13, 14]. Although to a lesser extent, men are at risk too for perinatal weight gain and increasing body mass index (BMI) [15, 16]. This may be the onset of lifelong overweight or obesity, its related non-communicable diseases and clinical and public health implications [17–20]. Thus, when having a first child, it is of uttermost importance to prevent undesirable weight gain in parents.

A behavioral aspect strongly associated with weight gain is eating behavior [21]. Favorable as well as unfavorable changes in dietary intakes throughout pregnancy until the postpartum period have been described, however, these changes are inconsistent and vary between women [22–24]. Moreover, eating behaviors may differ strongly between women and men [25]. Gaining insight into the determinants behind changes in eating behavior (what, when, how much and why people eat) of not only women, but also men during this lifechanging period is thus crucial. Understanding these changes is needed to provide expecting couples and first-time parents with purposeful eating advice and support. Some literature on determinants of eating behavior in the pregnancy and postpartum period is available. However, the focus often lies solely on weight management or on specific subgroups, such as pregnant women with gestational diabetes or obesity, African migrant pregnant women in high income countries, low-income women or women with increased cardiovascular risks [26–34]. Moreover, studies about determinants of eating behavior and lifestyle intervention studies during pregnancy and in the postpartum period including men are scarce but recommended. Involving the social environment of mothers, and not at the least the fathers, in the development and implementation of interventions could be an important asset [35–37]. A family-based focus should be considered, targeting the couple as a whole. Men can not only be essential for support, but also men themselves might be more motivated to improve (or maintain) their (healthy) eating behavior as a result of upcoming fatherhood [15, 16, 38].

Another important gap in the literature is the focus of most studies on eating behavior itself, whereas a focus on *changes* in eating behavior could be more effective to understand this transition phase. Delivering more specific knowledge is needed in the development of tailored interventions targeting unfavorable dietary changes. Few studies specifically focused on the *first* pregnancy- and postpartum period. Nevertheless, this period is crucial, as long-lasting changes in body weight and behavior as a result of the first pregnancy may have an impact on perinatal complications in subsequent pregnancies [39, 40]. Finally, as a child’s first year seems to be an overwhelming period for parents [41], understanding changes in eating behavior during these first challenging and demanding months is necessary. Therefore, this study aimed to identify determinants of changes in eating behavior across the transition to parenthood (i.e., during pregnancy up to 1 year postpartum) in both women and men.

## Methods

### Design and participants

A qualitative research design using focus group discussions was used. Participants were recruited through a purposeful snowball sampling strategy. Digital flyers and newsletters of organizations with a target audience of new parents or parents-to-be were sent to reinforce recruitment. Because of differences in energy metabolism and eating behaviors between women and men [25, 42], the research described in this article focused on male-female couples only. Moreover, the exclusion of same-sex couples allows a homogeneous comparable ‘partner group’. To this end, the following terminology will be used to refer to the participants: ‘(pregnant) women’/‘mothers’/ and ‘men’/‘fathers (−to-be)’. Two sets of focus groups were organized. For the first set focusing on changes during the pregnancy period, healthy women and men expecting their first child or having a first child less than 3 months old were recruited. For the second set focusing on changes during the postpartum period, first-time mothers and fathers having a first child of 3 months to 1 year old were recruited. Each participant could take part in only one focus group.

For both sets of focus groups, participants were recruited individually (e.g., when their partner did not want or could not participate) or as a couple. People without a condition that may influence their energy balance related behavior (e.g., diabetes) of any age, with any BMI and from any socio-economic status were eligible to participate. The healthcare system in Belgium is the same for everyone, regardless of the hospital or healthcare provider. The system is structured as such that everyone has access to information, customized and reimbursed care and parental leave during which an allowance is received. Employed women are entitled to a total of 15 weeks and women who work independently are entitled to a total of 12 weeks of maternity leave. Fathers are entitled to take 15 days of paternal leave. The recruitment of women and men up to 1 year postpartum enables to have a mix of participants who were still expecting, who were on parental leave and who already started working again. Both homogeneous (women- or men-only) and heterogeneous (mixed: couple-only groups and couples mixed with women/men without their partner) focus group discussions were conducted. This mix of focus group composition was done to ensure diversity of both interactions and opinions within and across focus groups. The aim was to recruit between 6 and 10 participants per focus group [43] from the five Flemish regions in order to ensure geographic distribution. For each focus group, an over-recruitment of one or two participants was pursued in case of ‘no-shows’ [43]. Focus groups were organized until saturation of information was reached, as sample size can never be pre-determined in qualitative research [43, 44].

### Ethics statement

Each focus group discussion started with an introduction on the purpose and content of the study while participants were asked to sign an informed consent. The protocol of the study and related documents were approved by the Medical Ethics Committee of the University Hospital (Vrije Universiteit Brussel, Brussels, Belgium). The study was conducted in compliance with the principles of the Declaration of Helsinki (current version), the principles of Good Clinical Practice (GCP) and in accordance with all applicable regulatory requirements.

### Procedure

All focus groups were conducted between March and June 2019 and were led by a female moderator (VV) and observer (Master student). To identify the point of theoretical saturation a preliminary content analysis was done shortly after each focus group discussion. When theoretical saturation was reached, one extra focus group discussion was performed to ensure true saturation. The moderator was trained by a senior researcher (TD) with extensive experience in qualitative research. All focus groups were organized in the evening, on a date and location convenient for the participants.

For each focus group a timing of 90 to 120 min was scheduled, including time for explaining the study purpose, signing the informed consent and answering a short questionnaire. This included questions on demographics (i.e., age and educational level: no degree, lower, secondary, college and university education), perceived health *(“How would you currently describe your health status in general?”* on a 5-point scale), perceived diet quality (*“To what extent do you feel you are eating healthy?”* on a 5-point scale), physical activity level (*“On how many days during the last week were you physically active for at least 30 minutes?”*), body weight (self-reported, in kg) and height (self-reported, in cm). After each focus group discussion, a body composition analysis, using a portable bio-electrical impedance analysis device (TANITA MC780SMA), was offered as an incentive. Not all participants made use of this possibility.

### Question guide

All focus group discussions were conducted following a semi-structured question guide (see Table 1). Being part of a larger study, the first part of the question guide focused on eating behavior, physical activity and sedentary behavior, a second part focused on intervention strategies. For the purpose of the current paper, only the questions and findings on changes in eating behavior and associated determinants were reported. The questions used to investigate these determinants were asked in two different ways (question 2 and 3) in order to trigger people to think thoroughly about the factors related with changes in their eating behavior.

**Table 1:** Semi-structured question guide, including questions related to changes in eating behavior

Questions about changes in eating behavior during pregnancy	Questions about changes in postpartum eating behavior
1. Think about the period before pregnancy/before the pregnancy of your partner; did your eating behavior change during pregnancy/during the pregnancy of your partner, and if yes how and to what extent?	1. Think about the period before pregnancy/before the pregnancy of your partner; did your eating behavior change since you became a mother/father, and if yes how and to what extent?
2. Which factors have caused these changes in eating behavior during pregnancy?	2. Which factors have caused these changes in eating behavior since your child was born?
3. Which of these factors do you think have had the biggest influence on these changes?	3. Which of these factors do you think have had the biggest influence on these changes?

### Data analysis

All focus group discussions were audio-taped and transcribed verbatim in Microsoft Word using Windows Media Player 12. NVivo 12 software was used for qualitative data analysis. A preliminary content analysis was done shortly after each focus group discussion (in order to determine theoretical data saturation), whereas the final data-analysis was performed after completing all the focus groups [45]. An inductive thematic approach was used to derive sub- and main categories from the data [46]. In a first step, all quotes derived from question 1 were used to provide a framework of changes in eating behavior. Quotes derived from questions 2–3 were taken together as quotes reflecting determinants causing changes in eating behavior. The codes were then grouped into units and subunits. This process was carried out by two researchers independently (VV & PD), ensuring reliability of data synthesis. These (sub) units were on their turn refined and renamed into a list of determinants. The final main- and subcategories were categorized through several discussion rounds involving three experts in the field of eating behavior (VV, MS & TD). Any doubts or disagreements were discussed until consensus was reached.

In order to systematically categorize and label the determinants, and to facilitate interpretation of findings, the Determinants of Nutrition and Eating (DONE) framework [47] was employed. Following the structure outlined in the socio-ecological DONE framework, each individual determinant (e.g., ‘hunger and satiety’) was assigned to a leaf-category (e.g., ‘food-related physiology’), within a stem-category (e.g., ‘biological’), and in turn to one of the main levels (e.g., ‘individual’). The full list of determinants and their categorizations can be found in Additional file 1. IBM SPSS Statistics 26 was used to analyze the quantitative data obtained from the questionnaires and to calculate descriptive statistics of the focus group sample.

## Results

### Study sample

A total of 13 focus groups were held, seven focusing on changes in energy balance related behavior (EBRB) during pregnancy and six focusing on changes in EBRB from birth up to 1 year postpartum. Being part of a larger study, time spent on discussing changes in EBRB ranged from 19 min to 55 min. Time spent on discussing dietary changes (part of the discussion on EBRB) ranged from 5 min to 20 min. A total of 98 participants were recruited, of which 24 did not show up or cancelled last minute. This resulted in a final sample of 74 participants, of which 36 were male and 38 were female. The number of participants per focus group ranged from three to eight. Seven focus groups consisted of a mix of couples (*n* = 14 couples) and one or more participants without their partner (women: *n* = 10, men: *n* = 3), two focus groups consisted of only couples (each 3 couples), two focus groups were women-only (each 4 women) and two were men-only (each 6 men). Sample characteristics are displayed in Table 2.

**Table 2:** Characteristics of the focus group participants (mean ± SD, %)

	Focus groups on changes in eating behavior during pregnancy		Focus groups on changes in postpartum eating behavior	
	Women	Men	Women	Men
Total sample (n)	22	20	16	16
Ethnicity (%)				
Caucasian	100	100	100	100
Age (years)	30.1 ± 2.5	31.6 ± 2.5	30.3 ± 2.0	31.7 ± 3.5
Self-reported pre-pregnancy BMI (kg/m^2^)	22.7 ± 3.1	24.0 ± 4.5	23.3 ± 4.7	24.4 ± 2.4
Respondents with a higher education (%)	81.8	75.0	93.8	87.5
Perceived health: Respondents reporting to be in good to very good health (%)	100.0	100.0	62.6	81.3
Respondents reporting healthy to totally healthy eating pattern (%)	77.3	80.0	93.8	62.6
Respondents reporting being active for at least 30 min/day for 5 days or more during the last 7 days (%)	49.9	45.0	6.3	37.5
% non-smokers (% ex-smokers)	100.0 (4.5)	100.0 (40.0)	100.0 (0.0)	100.0 (12.5)
Expecting parents (n)	15	14		
Gestational age (weeks)	28.4 ± 8.1	28.2 ± 8.6		
Parents with child (n)^a^	7	6	16	16
Age of the new-born (weeks)	9.6 ± 2.8	9.8 ± 5.2	34.8 ± 14.7	32.6 ± 15.3

^a^For the focus groups during pregnancy, both expecting parents, as well as parents with a first child less than three months old participated

### Changes in eating behavior during pregnancy and postpartum

Most participants described one or more changes in their eating behavior during either the entire or only during a specific period in the pregnancy or postpartum period. An overview of the changes is summarized in Table 3.

**Table 3:** Described changes in eating behavior during pregnancy vs. postpartum

During pregnancy	Postpartum
**1. Changes in food choice**	
Changes in preference and taste^a^:	None reported
- Food preference and aversion to specific foods	
- Taste preference and aversion e.g.*, sweet preferences*	
Changes in food choice related to convenience e.g.*, take-out food*	Changes in food choice related to convenience e.g.*, more food that is easy and fast to prepare*
**2. Changes in eating frequency and quantity**	
Increasing frequency of eating	Decreasing frequency of eating, more irregularity
Changes in quantity (smaller portions)^a^	Changes in quantity (skipping meals)
**3. Changes in time and place of eating**	
None reported	- Place e.g.*, more at the office, less in restaurants*
	- Time e.g.*, later in the evening*
**4. Changes in eating circumstances**	
None reported	e.g.*, eating more in a hurry*
**5. Changes in eating preparation**	
e.g.*, more attention for food safety*	e.g.*, preparing meals more in advance*
**6. Changes in dietary intake**	
- Healthier e.g.*, more vegetables*	- Healthier e.g.*, more fresh foods, more variety of vegetables and fruit*
- Unhealthier e.g.*, more sugary foods*	- Unhealthier e.g.*, more candy*
**7. Supplementation**	
Supplementation of vitamins	None reported

^a^changes mentioned by women only, all other changes were mentioned by both women and men

### Determinants of changes in eating behavior during pregnancy and postpartum

**Fig. 1:**
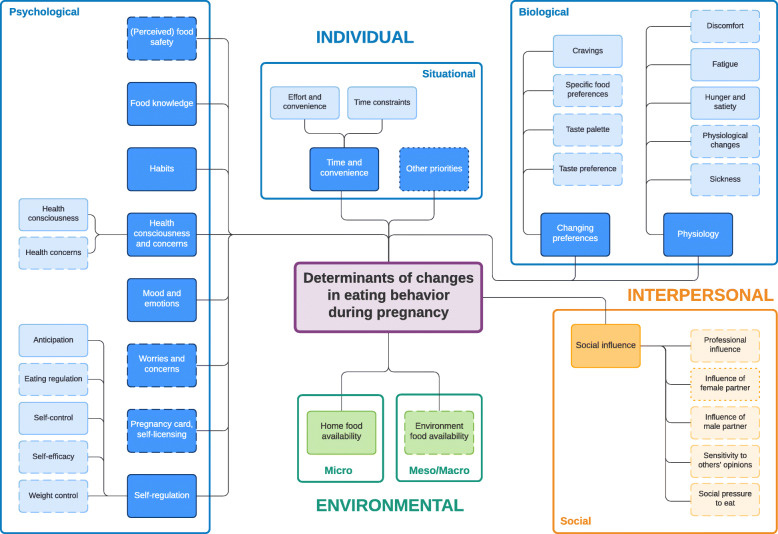
Determinants of changes in eating behavior during pregnancy. Full line: determinants mentioned by both women and men; dashed line: determinants mentioned only by women; dotted line: determinants mentioned only by men

**Fig. 2:**
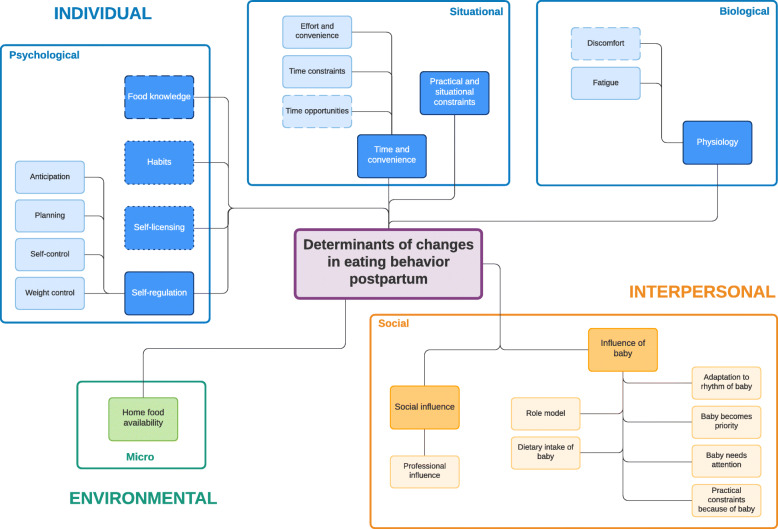
Determinants of changes in eating behavior from birth up to one year postpartum. Full line: determinants mentioned by both women and men; dashed line: determinants mentioned only by women; dotted line: determinants mentioned only by men

Following the principles of the DONE-framework [47] two frameworks were developed: (1) determinants of changes in eating behavior during pregnancy (Fig. 1) and (2) during the postpartum period (Fig. 2). Each framework consist of three main socio-ecological levels, i.e., the individual (subdivided into psychological, situational and biological), interpersonal (social) and environmental (subdivided into micro- and meso/macro) level. For each determinant described below, it is indicated whether it was mentioned during pregnancy and/or postpartum, after which each (sub) category is illustrated by the most appropriate quotes (Tables 4, 5, 6, 7 and 8).

### Individual level

#### Psychological

##### (Perceived) food safety – during pregnancy

Women changed their food choice because of (perceived) safety issues. This was either for the reason that specific ingredients should be avoided (e.g., smoked salmon) or the special attention needed to prepare foods such as washing vegetables thoroughly. Some participants were less strict [quote 1], while others were not even aware of food safety issues. Avoiding foods because of the lack of trust of information regarding ingredients or food safety issues when going out for dinner was also mentioned [quote 2].

##### Food knowledge – during pregnancy and postpartum

During pregnancy, both women and men mentioned conflicting advice and uncertainty about what is allowed or not, which often resulted in not eating questionable foods. A lack of information in terms of food safety issues was experiences, which made participants search for information on the internet [quote 3]. Postpartum, knowledge about the healthiness of foods was lacking [quote 4].

##### Habits – during pregnancy and postpartum

In Addition to their usual food preferences, participants reported newly developed eating habits throughout their pregnancy, which further persisted into postpartum. These new habits were mostly related to quantity (e.g., eating smaller portions) and eating frequency (e.g., snacking more in between meals) [quotes 5, 6]*.* Postpartum, one father noticed a change in his habits which he felt should be reversed again [quote 7].

##### Health consciousness and concerns – during pregnancy

Health consciousness, mentioned by women and men, comprises the attitudes of people about the healthiness of their diet and lifestyle. Food choices were based on the perceived healthiness of products for themselves and their baby [quote 8]. Women mentioned health concerns related to their eating behavior, such as being aware of eating too much sugar or drinking too much coffee [quote 9]*.* Some stated to do more efforts towards the end of pregnancy, while others’ motivation decreased.

##### Mood and emotions – during pregnancy

Mood swings had an influence on the eating behavior throughout pregnancy [quote 10]. The choice for convenience food was attributed to higher stress levels experienced by a father during the first trimester [quote 11].

##### Worries and concerns – during pregnancy

Women reported fear of excessive weight gain or concerns for the upcoming “glucose screening test” [quote 12]*.* Women were occupied with what they could eat and were afraid of becoming unhealthy or losing control of their eating behavior [quote 13, 14].

##### Self-licensing – during pregnancy and postpartum

Many pregnant women reported that they allowed themselves to eat more/differently (e.g., unhealthy snacking) because ‘they were pregnant’ and thus felt they deserved it. Some women described it as “pulling the pregnancy card”. Compensational behavior, not worrying about what they ate or their body weight, giving in faster to foods they liked, or eating bigger portions were often mentioned [quote 15]*.* Self-licensing in the postpartum period was only mentioned by men. Merely in a way of allowing themselves more (e.g., more comfort foods) because they were tired or just did not feel like cooking [quote 16]. Performing sports was also stated by men as an excuse to eat unhealthy [quote 17].

##### Self-regulation – during pregnancy and postpartum

Self-regulation was divided into the following subcategories: anticipation (both during pregnancy and postpartum), planning (postpartum), eating regulation (during pregnancy), self-control (both during pregnancy and postpartum), self-efficacy (during pregnancy), and weight control (both during pregnancy and postpartum). Anticipation was mentioned by both women and men, e.g., making sure healthy foods were available, or by cooking larger portions to have extra food available [quotes 18, 19]. Both women and men reported that, during the postpartum period, they tried to be at home together and be responsible for childcare and food preparation, or to make a grocery shopping list [quote 20]*.* Eating regulation, i.e., being able to control one’s own eating pattern, was described by some pregnant women. This included emotional eating, trying to eat healthy to control and balance what to eat [quote 21]*.*

Both women and men mentioned difficulties in terms of self-control (e.g., giving in more quickly to sweets, lack of self-control in the supermarket) during pregnancy and postpartum [quotes 22, 23]. One father explained that he did not purchase certain foods to avoid eating them. Men also mentioned being seduced easily by the (new, often unhealthy) preferences of their pregnant partner [quote 24]. Although challenging, both women and men during pregnancy realized they should change these new (unhealthy) behaviors.

Self-efficacy was stated as the difficulty to continue to eat healthy during pregnancy. Some pregnant women felt they could not do anything about the changes they experienced (e.g., being sick and as a result being able to drink only soda). Finally, weight control related quotes were reported during pregnancy and postpartum. Especially women expressed the desire to lose the pregnancy kilos [quotes 25, 26]. Some men said to be confronted with weight gain during pregnancy as well, which triggered them to change their eating behavior [quote 27].

**Table 4:** Quotes 1–27 about determinants of eating behavior on the individual (psychological) level

Subcategories of the psychological level	Quotes during pregnancy	Quotes postpartum
(perceived) food safety	Quote 1 (pregnant woman 6): “I was not allowed to eat raw vegetables, but I ate them anyway” Quote 2: (expecting father 1): “In the past, we occasionally went to eat somewhere, but during our pregnancy we didn’t, because we were not sure how the food was prepared.”	
Food knowledge	Quote 3 (expecting father 6): “I typically cook at home, and I also do the groceries, nowadays I just walk with my phone through the supermarket searching on the internet: ‘Pregnancy and this kind of food, is it allowed?’, ‘Is this good?’, is that good?’ (…) This is for me the biggest challenge, constantly looking for ‘Can she eat this?’, let’s quickly try to find out, never mind, let’s make something different today.”	Quote 4 (first-time mother 16): “Because you know more or less if something is (un) healthy, but you have to be very conscious about it, and if you have little time, it takes a lot of time to consciously make a choice when taking food from the fridge. Yes, my partner thinks that it is easy, but I find it sometimes difficult, because I was startled about ‘so that is also unhealthy?’, while I thought that it was still okay.”
Habits	Quote 5 (pregnant women 13): “My portions became smaller, − half the amount - since I was pregnant, because I just could not eat more, otherwise I became very nauseous. Now, my portions are still the same.” Quote 6 (pregnant women 11): “Before, I used to eat three meals a day, I’m not the kind of person that snacks a lot in between, unless occasionally a yoghurt or something in the evening, but not that often. Nowadays, I snack every day. I will have to lose that habit. (…) My partner also got used to snacking. If there are no snacks available, he remains hungry all the time.”	Quote 7 (first-time father 6): “These days it is easier to stop at a bakery for a pastry, while before I would just have baked a healthy apple cake myself, and I just have to get back into that habit, because in itself that is not a lot of work, but you have to just do it.”
Health consciousness and concerns	Quote 8 – Health consciousness (pregnant women 11): “I noticed that at first, I really tried hard since I thought, I’m pregnant so I have to make sure I eat healthily for my unborn child.” Quote 9 – Health concerns (pregnant women 4): “I kept on drinking coffee (…), but around 7 to 8 months into my pregnancy I noticed that the baby became more active after only 2–3 sips. And then I noticed ‘probably this is not too healthy’ (…), So then I said to myself ‘I will not drink coffee or caffeinated soda anymore’. (…) But then, when I had an energy dip at work, I was like, ‘I’m going to drink a coffee anyway’, and that caused the baby to become restless again. But then I thought ‘Yes, yes, it will …; there will not be such a harm anyway’.”	
Mood and emotions	Quote 10 (pregnant women 15): “I had a lot of mood swings. During the first three months it made me eat more chocolate and other snacks high in sugar.” Quote 11 (expecting father 17): “Perhaps also in the beginning, with those hormones that where more intense, sometimes this was a very stress period, and as a result maybe we ate more often fast food.”	
Worries and concerns	Quote 12 (pregnant women 6): “I didn’t want to eat anything, but truly nothing, I was really freaking out, because of fear for the ‘Glucose screening test’. Once the results came back negative, I was able to relax about it.” Quote 13 (expecting couple): Expecting father 4: “We were not planning to change our eating behavior, it is not that all of a sudden we would start eating meat because you are pregnant [to pregnant partner].” Pregnant woman 6 (partner of expecting father 4): “Indeed, and that’s why for me, those first months, I was panicking because I felt that my taste preference was changing so much that I thought ‘oh no, I am not going to be able to control this’.” Quote 14 (pregnant women 18): “That was a feeling for which I was really scared, to have these pregnancy cravings while sitting on the couch.”	
Self-licensing	Quote 15 (pregnant women 20): “During pregnancy, you are not allowed to drink alcohol anymore, and working out did not go so well anymore. My thinking was that if I can’t enjoy those things anymore, I will enjoy my food anyway. During my first trimester, every now and then, I did not pay attention anymore. (…) If I had a craving for a hamburger, I would have it.”	Quote 16 (first-time father 5): “Also the fatigue, it makes that you really feel like eating unhealthy foods.” Quote 17 (first-time father 6): “I know that it is not so disastrous because I work-out quite enough, so it is not so bad to eat unhealthy for a while.”
Self-regulation	Quote 18 - Anticipation (expecting father 5): “… we made sure we had a healthy spare meal somewhere.” Quote 21 – Eating regulation (pregnant women 11): “I noticed that if I forced myself to eat healthy, but I would not eat enough. And then I thought ‘it might be better to eat unhealthy things, rather than not eating enough’.” Quote 22 – Self-control (pregnant women 3): “I am eating more fruits, but sometimes I am more inclined to eating sweets, whereas before I could easily decline unhealthy food, now it is more difficult.” Quote 24 – Self-control: (expecting father 7): “I am not going to say ‘no’ if my wife proposes to order fast food.” Quote 25 – Weight control (pregnant women 4): “Yes, we ate healthier, (…), and also less sugar-rich food, so that weight gain was mainly due to the baby, and not because of paying less attention to your food.”	Quote 19 – Anticipation (first- time mother 7): “I have to pump breastmilk, so I am not able to join my colleagues for lunch, so I always have to make sure that I have healthy food with me.” Quote 20 – Planning (first-time father 16): “We are planning a lot more our meals, from Sunday to Wednesday, and from Wednesday to Friday. (…) Before we used to call each other in the evening ‘Can you pick up groceries?’, while now we are actually combining these things to make shopping more efficient.” Quote 23 – Self-control (first-time father 15): “All those birth visits during those first weeks, did not help much either: so many chips and the cookies, and I do know myself …” Quote 26 – Weight control (first-time mother 5): “I didn’t want to become a mom who would gain 5 k with every pregnancy, and end up 20 k heavier, I really didn’t want that. So for me it was very important to return to my initial weight.” Quote 27 – Weight control (first-time father 5): “The fact that I already had gained a lot during the pregnancy of my wife, it was so confronting, that it triggered something in me. I believe that I cook more now than before pregnancy.”

Quotes from people who participated in the first set of focus groups were identified as ‘pregnant women x’ or ‘expecting father x’, even though some already delivered at the moment of the focus group discussion. Quotes from people who participated in the second set of focus groups were identified as ‘first-time mother x’ or ‘first-time father x’

#### Situational

##### Time and convenience – during pregnancy and postpartum

Many participants did not feel like cooking or spending time preparing healthy meals, as it required too much effort or because they were too tired. This resulted often in take-out food or unhealthy, convenient, and easy to prepare foods [quote 28–30]. Some couples mentioned they tried to eat healthy without extensive effort by ordering meal boxes. A lack of time to eat was quoted during the postpartum period [quote 31]*.* In contrast with mentioned lack-of-time barriers, some mothers also experienced time opportunities when they were still at home during their maternity leave, making it possible to cook healthy [quote 32].

##### Other priorities – during pregnancy

Enjoying free time as much as possible prior to becoming a parent was reported as a priority over cooking by one of the male participants [quote 33]*.* Postpartum, the baby became a priority over preparing healthy meals (see further under “0: Interpersonal level”).

##### Practical and situational constraints – postpartum

In the postpartum period, changes were also attributed to practical and situational constraints, such as doing a lot of household tasks or unpredictable time schedules [quote 34].

**Table 5:** Quotes 28–34 about determinants of eating behavior on the individual (situational) level

Subcategories of the situational level	Quotes during pregnancy	Quotes postpartum
Time and convenience	Quote 28 – Time constraints (expecting father 3): “I was less eager to cook for one hour, if it could be done in half an hour, it was half an hour.”	Quote 29 – Effort and convenience (first-time mother 7): “I used to be conscious about food choices before my pregnancy, but now, I notice I just eat what I want. Before, I never ate bread, but now I eat it every day because it is convenient. Whereas before I took time to prepare healthy meals containing vegetables, and I ate a salad during lunch …” Quote 30 – Time constraints (first-time father 10): “Usually, I try to make oatmeal with fruit in the morning, but sometimes there is no time and then I prepare cornflakes for us.” Quote 31 – Time constraints (first-time mother 7): “During these three months that I was at home, I ate with my partner to ensure I would have my breakfast, because otherwise, some days I only had time at 4 pm to quickly grab a snack.” Quote 32 – Time opportunities (first-time mother 5): “I had 5 months of maternity leave and thus I had the time to focus on healthy meals. If you go to work, then you don’t have time for this.” (talking about efforts to eat healthy to come back to pre-pregnancy weight).
Other priorities	Quote 33 (expecting father 9): “I had the impression that I just did not want to cook as often anymore, it took so long (…) because I thought: ‘now I still have quite a lot of free time, and I want to take advantage of that, because once the baby is here we will be busy anyway with cooking.”	
Practical and situational constraints		Quote 34: (first-time father 16): “I think you go through a lot of unpredictable things you need to adjust to, especially in the first months”

Quotes from people who participated in the first set of focus groups were identified as ‘pregnant women x’ or ‘expecting father x’, even though some already delivered at the moment of the focus group discussion. Quotes from people who participated in the second set of focus groups were identified as ‘first-time mother x’ or ‘first-time father x’

#### Biological

##### Changing preferences – during pregnancy

Changes in taste preference (e.g., more sweet preferences) or changes in taste palette were described [quote 35]. Food preferences that changed during pregnancy often reverted after a while [quote 36]*.* Finally, different kinds of cravings were quoted, such as the urge for chocolate and foods high in sugar, especially at the start of pregnancy. One father-to-be talked about the vicious circle of eating greasy foods as a consequence of the cravings of his partner for pizza [quote 37].

##### Physiology – during pregnancy and postpartum

Discomforts and sickness (e.g., heartburn, nausea) impacted women’s eating behavior, especially at the beginning of their pregnancy [quote 38]. To avoid these problems, some women did not eat certain sorts of food (e.g., candy) anymore, while others started eating specific foods (e.g., mashed potatoes, rice cakes, cookies). The described changes were thus related to both improvement or impairment of the quality of their eating pattern [quotes 39]. Besides eating (or avoiding) specific foods, eating more frequently and decreasing portion sizes were other preventative approaches to avoid a hungry/sick feeling. Eating throughout the day, being hungry the feeling of insatiability, or not being able to find which kind of foods may give a saturated feeling were quoted [quote 40, 41]. Physiological changes including hormonal influences but also the feeling of a different (i.e., pregnant) body influenced women’s eating behavior. The changing body geometry or pregnancy-related dizziness made things physically difficult, e.g., to shop groceries or cooking [quote 42]. Some men were getting used to eating more frequently together with their partner, which impacted their satiation [quote 43].

Fatigue (i.e., lack of energy, being too tired, mental fatigue) was quoted by the couples as an important determinant of changes in eating behavior [quote 44]. Especially postpartum quotes about being exhausted and fatigue were equally mentioned by women and men. As a result, people lacked energy to cook or prepare meals [quote 45] or considered this as a reason to choose different kinds of food such as comfort foods or caffeine-rich foods [quote 46].

**Table 6:** Quotes 35–46 about determinants of eating behavior on the individual (biological) level

Subcategories of the biological level	Quotes during pregnancy	Quotes postpartum
Changing preferences	Quote 35 – Taste palette (pregnant women 6): “I imagined how something tasted so I felt like eating it, but when I tasted it I thought ‘Oh no, I actually don’t want to eat this at all.’.” Quote 36 – Specific food preferences (pregnant women 5): “I used to eat yoghurt with fruit every day, but while pregnant I had no desire for it anymore. Nowadays, I eat several pieces of fruit in a day without effort. Eating enough vegetables is sometimes difficult though. During the first 3 months, I ate almost nothing, ‘a banana then’ I thought, ‘I have to eat some kind of fruit’, and then I only ate bananas.” Quote 37 – Cravings (expecting father 3): “And thing with these ‘greasy foods’ is that if you eat it more often, you want it again more often. (…) And then you notice, the more often you eat something like that, the more often you feel like eating it.”	
Physiology	Quote 38 – Sickness (pregnant women 21): “Now that the nausea is gone I eat again as prior to my pregnancy.” Quote 39 – Discomfort (pregnant women 5): “It’s not unhealthier in all aspects, it is actually healthier in some aspects, because I really liked sweets in the evening, not anymore now because if I eat sweet foods in the evening, I get a lot of gastric issues.” Quote 40 – Sickness (pregnant women 12): “In the beginning I was not really nauseous, but I constantly had the feeling that I had to eat to avoid it When I go to work now, I make sure to take a lot of snacks with me.” Quote 41 – Hunger and satiety (pregnant women 13): “My portions shrunk by half of the portions before I was pregnant, because I couldn’t eat more.” Quote 42 – Physiological changes (expecting father 7): “It became more uncomfortable for her [about pregnant partner] to cook.” Quote 43 – Hunger and satiety (expecting couple): Pregnant women 11: “And he as well [about partner] got used to eating more snacks throughout the day, otherwise he’s hungry all the time.” Expecting father 13 (partner of pregnant women 11): “Yes, especially now towards the end of her pregnancy I am constantly hungry. I normally do not eat breakfast, usually I get up and go to work, and then I eat something during the day. But since I am more hungry now, I started to eat in the morning.” Quote 44 – Fatigue (expecting father 9): “I cook more often compared to the past because she [about pregnant partner] is more tired when she comes home from work and takes a break while I cook. This did not happen before.”	Quote 45 – Fatigue (first-time mother): “You just don’t have the energy to cook after work and handling with the baby stuff.” Quote 46 – Fatigue (first-time father 1): “These first months our son almost didn’t sleep and I fell asleep at work. Therefore I took chocolate or caffeinated soda, which I otherwise never do, but back then I did, because otherwise I just couldn’t stay awake.”

Quotes from people who participated in the first set of focus groups were identified as ‘pregnant women x’ or ‘expecting father x’, even though some already delivered at the moment of the focus group discussion. Quotes from people who participated in the second set of focus groups were identified as ‘first-time mother x’ or ‘first-time father x’

### Interpersonal level

#### Social influence – during pregnancy and postpartum

Professional influence impacted women’s eating behavior, both positively and negatively. Some pregnant women were reassured or warned by a doctor or dietitian about their eating pattern and weight gain [quote 47]. One pregnant woman explained she was warned by her gynaecologist to be at risk for developing gestational diabetes, without doing a proper dietary assessment. The woman explained that she and her partner experienced this as “unsubstantiated and threatening”. One mother talked about the support during the postpartum period (e.g., friends bringing over healthy dishes), while this was not the case during pregnancy. Women experienced social pressure to eat more throughout pregnancy. This negatively perceived social influence mostly came from family and colleagues [quote 48], but from the partner as well. One father-to-be stated to give bigger portions to his pregnant partner because *“she is now one and a quarter”*. It was mentioned, though, that sensitivity to other opinions may in- or decrease susceptibility to this kind of social pressure.

Participants, however, described positive influences of the male partner too, e.g., by cooking, or by not drinking alcohol to support his pregnant partner [quote 49]. One father-to-be discouraged his pregnant partner from eating chocolate, but as a result, she started snacking secretly. Another father-to-be tried to be supportive by eating more and gaining weight so that she would not feel guilty after the gynaecologist told her she gained too much weight. Many men adapted their eating patterns to the preferences and choices of their pregnant partner [quote 50]. This also increased the consumption of snacks in between meals, which were more available, or the food their pregnant partner did not like or want to eat [quote 51, 52]. Some men increased their alcohol consumption (e.g., because they had always someone to drive the car when going out), whereas others decreased their alcohol consumption. This was done as an expression of solidarity or as it was no longer possible to share alcoholic drinks with their pregnant partner [quote 53].

In the postpartum period, professional influence in terms of maternity help shortly after birth was mentioned, which allowed couples to receive external help including meal preparation. Furthermore, the food recommendations for the new-born from the government made participants try more vegetables than they were used to [quote 54].

#### Influence of the baby – postpartum

Adaptations to the rhythm of the baby were mostly related to a change in time when participants eat, dependent on the mood or the (breast) feeding time/rhythm of the baby [quote 55]. As a result, participants stated it was not easy to find a new rhythm and they had to put themselves aside. In case the baby needed a lot of attention (e.g., when the baby cries), participant explained to be prevented from eating in a relaxed way. This caused them to choose foods they can eat while holding the baby or to quickly finish their meals [quote 56]. Sometimes participants forgot to eat [quote 57]. Parents additionally mentioned practical constraints related to the baby. Dinner time delayed because of the difficulty to eat in a relaxed way when the baby wanted to eat along, or when meals were interrupted because one has to guard a crawling baby. The latter made cooking difficult, especially if one is home alone with the baby. Other practical issues were cited by breastfeeding mothers, as pumping breastmilk is time-consuming, or they were restricted in what they could eat [quote 58]. Finally, for many parents, spending time with the baby became a priority over preparing meals [quote 59].

On the other hand, both mothers and fathers stated positive influences of the baby. Once the baby was aware of what they did, parents felt they had to act as a role model (e.g., avoid drinking soda or eating snacks in the evening) [quote 60]. The dietary intake of the baby had a positive impact on the fruit and vegetable consumption of the parents [quote 61]. In the postpartum period, parents often adapted their eating pattern to the variety of vegetables the baby had to try (e.g., fennel, parsnip, pumpkin etc.).

**Table 7:** Quotes 47–61 about determinants of eating behavior on the interpersonal level

Categories of the interpersonal level	Quotes during pregnancy	Quotes postpartum
Social influence	Quote 47 – Professional influence (pregnant women 19): “My gynaecologist warned me because I gained too much weight over a period of five months.” Quote 48 – Social pressure to eat (pregnant women 19): “What also affected me were family and colleagues: ‘eat another piece of cake, it won’t hurt you, grab some more chocolate …’.” Quote 49 – Influence of male partner (expecting father 8): “I did a lot of efforts to cook tasty, but I had the impression that the less taste there was in the food, the better she liked it. So in the end, I just made a dish for her with less herbs, and something spicier for myself.” Quote 50 – Influence of wife (expecting father 7): “She only wanted to eat pizza and pasta, so I ended up eating it too.” Quote 51 – Influence of wife (pregnant women 11): “I think for you [to male partner], the main issue was having food nearby. If there were cookies for me on the table, you automatically started to eat them as well.” Quote 52 – Influence of wife (expecting father 4): “Then I started eating her portions, and it all went wrong.” (talking about left over food because of the changing preferences of pregnant partner) Quote 53 – Influence of wife (expecting father 11): “I can only think of alcohol consumption. Because she was not allowed to drink alcohol during pregnancy. (…) We did not longer have that open bottle of wine during the weekends.”	Quote 54 – Professional influence (first- time father 5): “We started eating a larger variety of vegetables after receiving a list from a parent support organization containing advise on what she [about daughter] should eat.”
Influence of baby		Quote 55 – Adaptation to rhythm of baby (first-time mother 11): “We used to eat quite early in the evening, when he [about partner] came home, because he cooks. But now we either eat before or after the bottle of our baby. It also depends on his [about baby] mood and if we can leave him alone for a while.” Quote 56 – Baby needs attention (first-time mother 13): “If I hear the baby, I think ‘Oh no, he needs something’, and then I eat very quickly so I can take care of him or comfort him.” Quote 57 – Baby needs attention (first-time mother 16): “I think less about food, because I am busy with everything else all the time. This makes that I sometimes forget, ‘Oh right, we still have to eat as well’.” Quote 58 – Practical constraints because of baby (first-time mother 15): “I breastfeed and this causes me to not eat certain things because of cramps [for the baby].” Quote 59 – Baby becomes priority (first-time father 16): “Not necessarily lack of time to cook, it is more a time management issue, you want much more time … If you have an hour you would rather play 55 min with your child, and spent 5 min to prepare food.” Quote 60 – Role model (first-time mother 14): “For example, not having soft drinks is especially an issue for me, but I need to be a good example to him [the baby].” Quote 61 – Dietary intake of baby (first-time mother 12): “I do eat more fruits and vegetables since he [baby] has to eat fruits and vegetables.”

Quotes from people who participated in the first set of focus groups were identified as ‘pregnant women x’ or ‘expecting father x’, even though some already delivered at the moment of the focus group discussion. Quotes from people who participated in the second set of focus groups were identified as ‘first-time mother x’ or ‘first-time father x’

### Environmental level

#### Micro

##### Home food availability – during pregnancy and postpartum

Different reasons (e.g., physically difficult to go shopping, dizziness) made grocery shopping more complicated for pregnant women, and thus, some participants stated to have insufficient healthy food available at home. Both women and men also stated that it was not that easy anymore to resist buying unhealthy foods (e.g., because they were hungry while doing groceries) [quote 62]. Having (un) healthy foods at home was crucial for their own consumption (e.g., snacks during birth visits versus the availability of healthy foods for the baby) [quote 63]. One couple even took a subscription for vegetables from a farmer, which forced them to cook and eat healthy.

#### Meso/macro

##### Environment food availability – during pregnancy

Some pregnant women mentioned the difficulty to find healthy alternatives for foods when eating out or ordering delivery because of food safety issues [quote 64].

**Table 8:** Quotes 62–77 about determinants of eating behavior on the environmental level

Categories and subcategories of the environmental level	Quotes during pregnancy	Quotes postpartum
Micro		
Home food availability	Quote 62 (pregnant women 7): “We only eat junk food if we buy it. Not buying it helps, but that has not worked out very well.”	Quote 63 (first-time mother 12): “Nowadays, we always make sure that there are fruits and vegetables available, because otherwise there is nothing to feed the baby.”
Meso/Macro		
Environment food availability	Quote 64 (expecting father 19): “If we went out for dinner, more than half of it was not an option due to food safety issues.”	

Quotes from people who participated in the first set of focus groups were identified as ‘pregnant women x’ or ‘expecting father x’, even though some already delivered at the moment of the focus group discussion. Quotes from people who participated in the second set of focus groups were identified as ‘first-time mother x’ or ‘first-time father x’

## Discussion

The aim of this study was to identify determinants of changes in eating behavior during pregnancy and postpartum for both first-time mothers and fathers. Following the DONE-framework, explaining determinants shaping nutrition and eating behavior [47], determinants were defined at three different levels, namely the individual, interpersonal and environmental level.

When interpreting the results, a strong interaction between the determinants of the different levels and sublevels came forward. The majority of determinants identified by this research can be classified within the individual level. This can be attributed to the physical and psychological changes primiparous women go through [1, 5, 8, 9]. Already during the preconception phase, women experience challenges such as stress and anxiety concerning the upcoming pregnancy impacting their health related attitudes [48]. The present study shows that these aspects seem to sustain during pregnancy. Psychological determinants such as beliefs and emotions have been identified as core drivers of (changes in) eating behavior, both in the pregnancy and postpartum period [1, 21, 48]. This often results in reduced intake of foods which may harm women’s pregnancy, rather than increased intake of foods providing key nutrients required for pregnancy [49]. The psychological determinants are likely to be linked with the identified biological determinants. In accordance with previous studies, the present study describes the undesirable impact of cravings, specific food preferences, physiological changes, etc. on changes in eating behavior of pregnant women [50, 51]. However, except for fatigue issues, the biological impact on eating behavior becomes negligible during postpartum, which is confirmed by other research [41]. The psychological and biological determinants can also be linked with situational barriers expecting and first-time parents experience (e.g., time constraints), as well as with environmental factors (e.g., the (un) availability of (un) healthy foods at home). Unavailability of healthy foods and availability of unhealthy foods, on the other hand, may equally be the result of barriers such as lack of food knowledge (food literacy) or the absence of self-regulation skills. This highlights the importance of nutritional education and training of self-management skills among this population. Improvement of these skills should be part of future interventions.

Another illustration of interacting determinants can be found when the baby is born. The birth of ababy hugely changes the living environment together with the new role and responsibilities parents experience [30, 52–54]. This may result in positive changes in terms of food choices (e.g., the willingness to be a role model), and negative changes in terms of eating occasion and time of eating (e.g., skipping meals, eating less relaxed and more in a hurry). Furthermore, a lack of effort (i.e., situational) to prepare healthy foods may be present as a result of fatigue (i.e., biological) and the attention the baby calls for (i.e., social). It is clear from the present but also other research that changes in eating behavior among women and men transitioning into parenthood are very individual-specific and determined by multiple factors at the same time [55]. In turn, these may have an impact on excessive weight gain and weight control issues during pregnancy and postpartum [21, 22, 56]. A personalized approach with tailored advise depending on the (interplay between) barriers and motivators is recommended.

When developing interventions, targeting women solely is not recommended. Even though most determinants described in this research reflect women’s experiences, men too experience significant changes in eating behavior during the pregnancy and the postpartum period. Men’s eating behavior is influenced by their pregnant partner, which often made them crave for specific foods because of her new preferences. Men in addition attribute changes in their eating behavior to a physiological impact such as fatigue and to situational determinants such as time constraints. Even though fathers(−to-be) may benefit from it, they are often neglected and experience a lack of (professional) support, a finding also supported by others [57]. As fathers-to-be may play an important role in supporting their pregnant partner, focus should go to the couple as a whole. It has been shown that family-based approaches targeting health behavior generally result in higher effectiveness compared to individual-targeted strategies [37].

Improving knowledge and awareness in expecting and first-time parents about the psychological and physical barriers, about food safety issues and the healthiness of the diet are important challenges to focus on when developing interventions. Unfortunately, the current study suggests that nutrition information during pregnancy is often perceived as confusing and overwhelming, which resonates with earlier research findings [58, 59]. Frustrations about the lack of food knowledge and dissuaded foods (in terms of food safety) may result in anxiety, fear and guilt, or in food indulgence to compensate certain restrictions. This may impact how expecting and first-time parents make certain food choices [58, 59]. It is moreover clear from our data that pregnant women experience social pressure and support from colleagues, family and friends, but equally from healthcare providers. Their important role in providing nutritional support and education has been extensively described in populations of pregnant women [50, 58, 60–62]. But, there is a need to educate healthcare providers themselves in order to give adequate, individualized and practical nutritional support and assistance [55, 60, 61]. The importance of the social environment, including the father, has also been highlighted in other studies [35, 59]. A family-based and multidisciplinary approach including specific training of healthcare providers to give tailored nutritional support is recommended. Also, by using a family-based approach, parents’ awareness on how the social environment (including themselves as a couple) may influence their eating behavior should be raised.

The participants in our study did not mention any determinants at the community and policy levels. It may be that the community and policy level play a more inferior role concerning *changes in* eating behavior during the transition to parenthood. In contrast, the ecological model of health behavior [63, 64] shows the importance of these levels when focusing on the behavior *as such*. Hence, these levels should equally be taken into account when developing and implementing interventions, integrating dietary guidelines for expecting and first-time parents. Although governmental organizations in Belgium provide nutritional guidelines, integration of these guidelines in the existing care pathways of women (let alone men) is missing during pregnancy and postpartum. This might be an explanation for the lack of (reliable) information and food knowledge, lack of knowledge to deploy self-regulation skills and the health concerns our participants experienced. There is an urgent need for clear, accurate and homogeneous guidance and support from governmental organizations on training and providing a platform enabling caregivers to support healthy eating behavior in expecting and first-time parents. Moreover, this study is a starting point. We invite other researchers to further investigate determinants of changes in eating behavior in different (sub) populations and within different family forms (e.g., single parents, same-sex parents). Future studies may add specific determinants to the frameworks. Additionally, confirmation of cause-and-effect relationships of the identified determinants within a quantitative longitudinal study is needed.

### Strengths and limitations

We consider the qualitative approach using focus group discussions a first strength of this study. Focus groups are a naturalistic (i.e., close to everyday conversation) approach and include dynamic group interactions, which provides in-depth insights and encourages participants to explore and clarify perspectives [45, 65]. Second, in order to increase internal validity two researchers with extensive experience in qualitative research and eating behavior supervised the data-analysis. Third, participants were recruited from five regions in Flanders (the Northern part of Belgium) which ensures diversity in geographic distribution. Moreover, the average age the women in our study (30.3 years old) is in line with the average age Belgian women having their first baby (29.3 years old) [66]. Fourth, both mixed and same-sex focus groups were used to enable a variety of opinions and interactions during the discussions. Including couples allows to take up the perspective of fathers (−to-be) in order to facilitate and integrate a family-based approach in future interventions. Lastly, by using two sets of focus groups with a great diversity of participants, ranging from being in the first trimester of pregnancy up to 1 year postpartum, recall bias of participants was reduced to a minimum. This made it possible to investigate determinants of changes in eating behavior throughout the entire period under study. There are some limitations to this study that are important to mention. First, although the purpose of the study was explorative in nature, we might have missed determinants due to selection bias. A rather homogeneous sample of Caucasian, mostly higher educated, and physically healthy participants was included. The inclusion of mainly health-conscious participants is a common limitation in studies investigating health behavior [67]. As a result our sample may not be representative for the entire population of first-time parents, possibly biasing our findings. For example, in the postpartum period, ‘cost’ might be a determinant explaining changes due to additional costs related to the baby (especially in people with a lower socio-economic status), but this did not came forward in our focus group discussions. We did not specifically question whether participating parents were single or not, while this would have given us extra insights into the data. The focus and importance might thus have been slightly different for specific (e.g., single parents, same-sex parents) or vulnerable populations (e.g., teenage parents, parents with low socio-economic status, parents without the Belgian nationality or a migrant background) or families with more children. Moreover, cross-country differences in eating behavior barriers during pregnancy and postpartum have been described [55]. This is worth noting as this might have implications for the generalizability of the findings. Second, although we added sex-specific information to our findings, our qualitative research design does not allow for statistical inferences, and thus it can be a matter of chance that some determinants were quoted by women or men only. Hence, these should not be interpreted per se as determinants exclusively applicable for women and/or men. This sex-specific information should thus be interpreted with great caution. Future research should further investigate the importance of these determinants in different (sub) populations of expecting and first-time parents, taking into account the difference in importance of each determinant between women and men.

## Conclusion

This study reveals the complexity of determinants of changes in eating behavior in pregnancy and postpartum in both women and men, situated at the individual, social and environmental levels. Individual determinants are recognized as main barriers or motivators to practice healthy eating. Because of the interaction between the different factors and levels of determinants, a focus on the interplay of psychological, biological, factors and situational difficulties is needed during pregnancy. Postpartum, a focus on how to cope with the new balance of maintaining one’s own health and taking care of the baby is recommended. In addition, individualized support on how to improve self-regulation skills or cope with situational constraints is needed. The present findings and developed frameworks form a solid base which can be used by gynaecologists, midwives and dietitians involved in pregnancy/postpartum care to provide dietary guidance and support for expecting and first-time parents. Finally, our frameworks can be used for the development of family-based interventions aiming to improve couple’s eating behavior in the lifechanging phase of getting a first child.

## Supplementary Information

**Additional file 1.** Categorization (leaf-category, stem-category and main level) of determinants of changes in eating behavior during pregnancy and postpartum.

## Data Availability

Audio tapes and transcribed interviews of the study can be retrieved through the corresponding author on reasonable request.

## Abbreviations

BMI: Body mass index
EBRB: Energy balance related behavior
DONE: Determinants of Nutrition and Eating
