# Supplementary material for: Determinants of changes in women’s and men’s eating behavior across the transition to parenthood: a focus group study
Source: Int J Behav Nutr Phys Act. 2021 Jul 12;18:95. doi: 10.1186/s12966-021-01137-4 (PMC8276457; doi:10.1186/s12966-021-01137-4)
Supplement: Supplementary file 1 — Additional file 1. Categorization (leaf-category, stem-category and main level) of determinants of changes in eating behavior during pregnancy and postpartum. [file 12966_2021_1137_MOESM1_ESM.docx]

**Additional file 1:** Categorization (leaf-category, stem-category and main level) of determinants of changes in eating behavior during pregnancy and postpartum

| Determinant | Leaf-category |  | Stem-category | Main level | Determinant | Leaf-category | Stem-category |  | Main level |  |
| --- | --- | --- | --- | --- | --- | --- | --- | --- | --- | --- |
| **DURING PREGNANCY** |  |  |  |  | **AFTER PREGNANCY** |  |  |  |  |  |
| **(Perceived) food-safety** | Food beliefs |  | Psychological | Individual |  |  |  |  |  |  |
| **Food knowledge** | Food knowledge, skills and abilities |  | Psychological | Individual | **Food knowledge** | Food knowledge, skills and abilities | Psychological |  | Individual |  |
| **Eating habits** | Food habits |  | Psychological | Individual | **Eating habits** | Food habits | Psychological |  | Individual |  |
| **Health consciousness and concerns** | Health cognitions |  | Psychological | Individual |  |  |  |  |  |  |
| Health consciousness | Health cognitions |  | Psychological | Individual |  |  |  |  |  |  |
| Health concerns | Health cognitions |  | Psychological | Individual |  |  |  |  |  |  |
| **Mood and emotions** | Mood and emotions |  | Psychological | Individual |  |  |  |  |  |  |
| **Worries and concerns** | Worries and concerns |  | Psychological | Individual |  |  |  |  |  |  |
| **Self-licensing (pregnancy card)** | Self-regulation |  | Psychological | Individual | **Self-licensing** | Self-regulation | Psychological |  | Individual |  |
| **Self-regulation** | Self-regulation |  | Psychological | Individual | **Self-regulation** | Self-regulation | Psychological |  | Individual |  |
| Anticipation | Self-regulation |  | Psychological | Individual | Anticipation | Self-regulation | Psychological |  | Individual |  |
| Eating regulation | Eating regulation |  | Psychological | Individual | Planning | Self-regulation | Psychological |  | Individual |  |
| Self-control | Self-regulation |  | Psychological | Individual | Self-control | Self-regulation | Psychological |  | Individual |  |
| Self-efficacy | Personality |  | Psychological | Individual | Weight control | Weight control cognitions and behaviors | Psychological |  | Individual |  |
| Weight control | Weight control cognitions and behaviors |  | Psychological | Individual |  |  |  |  |  |  |
| **Time and convenience** | Situational and time constraints |  | Situational | Individual | **Time and convenience** | Situational and time constraints | Situational |  | Individual |  |
| Effort and convenience | Situational and time constraints |  | Situational | Individual | Effort and convenience | Situational and time constraints | Situational |  | Individual |  |
| Time constraints | Situational and time constraints |  | Situational | Individual | Time constraints | Situational and time constraints | Situational |  | Individual |  |
| **Other priorities** | Situational and time constraints |  | Situational | Individual |  |  |  |  |  |  |
|  |  |  |  |  | **Practical and situational constraints** | Situational and time constraints | Situational |  | Individual |  |
| **Changing preferences** | Sensory perception |  | Biological | Individual |  |  |  |  |  |  |
| Cravings | Sensory perception |  | Biological | Individual |  |  |  |  |  |  |
| Specific food preferences | Sensory perception |  | Biological | Individual |  |  |  |  |  |  |
| Taste palette | Food-related physiology |  | Biological | Individual |  |  |  |  |  |  |
| Taste preference | Sensory perception |  | Biological | Individual |  |  |  |  |  |  |
| **Physiology** | Physiology |  | Biological | Individual | **Physiology** | Physiology | Biological |  | Individual |  |
| Discomfort | Physiology |  | Biological | Individual | Discomfort | Physiology | Biological |  | Individual |  |
| Fatigue | Physiology |  | Biological | Individual | Fatigue | Physiology | Biological |  | Individual |  |
| Hunger and satiety changes | Food-related physiology |  | Biological | Individual |  |  |  |  |  |  |
| Physiological changes | Physiology |  | Biological | Individual |  |  |  |  |  |  |
| Sickness | Physical health |  | Biological | Individual |  |  |  |  |  |  |
| **Social influence** | Social influence |  | Social | Interpersonal | **Social influence** | Social influence | Social |  | Interpersonal |  |
| Influence of male partner | Social influence |  | Social | Interpersonal | Professional influence | Social influence | Social |  | Interpersonal |  |
| Influence of pregnant wife | Social influence |  | Social | Interpersonal |  |  |  |  |  |  |
| Professional influence | Social influence |  | Social | Interpersonal |  |  |  |  |  |  |
| Sensitivity to other opinions | Social influence |  | Social | Interpersonal |  |  |  |  |  |  |
| Social pressure to eat | Social influence |  | Social | Interpersonal |  |  |  |  |  |  |
|  |  |  |  |  | **Influence of baby** | Social influence | Social |  | Interpersonal |  |
|  |  |  |  |  | Adaptation to rhythm of baby | Social influence | Social |  | Interpersonal |  |
|  |  |  |  |  | Baby becomes priority | Social influence | Social |  | Interpersonal |  |
|  |  |  |  |  | Baby needs attention | Social influence | Social |  | Interpersonal |  |
|  |  |  |  |  | Dietary intake of baby | Social influence | Social |  | Interpersonal |  |
|  |  |  |  |  | Practical constraints because of baby | Social influence | Social |  | Interpersonal |  |
|  |  |  |  |  | Role model | Social influence | Social |  | Interpersonal |  |
| **Home food availability** | Home food availability |  | Micro | Environment | **Home food availability** | Home food availability | Micro |  | Environment |  |
| **Environment food availability** | Environment food availability and accessibility |  | Meso/Macro | Environment |  |  |  |  |  |  |
